# Early sex-dependent differences in metabolic profiles of overweight and adiposity in young children: a cross-sectional analysis

**DOI:** 10.1186/s12916-023-02886-8

**Published:** 2023-05-09

**Authors:** Sandi M Azab, Meera Shanmuganathan, Russell J de Souza, Zachary Kroezen, Dipika Desai, Natalie C Williams, Katherine M Morrison, Stephanie A Atkinson, Koon K Teo, Meghan B Azad, Elinor Simons, Theo J Moraes, Piush J Mandhane, Stuart E Turvey, Padmaja Subbarao, Philip Britz-McKibbin, Sonia S Anand

**Affiliations:** 1 Department of Medicine, McMaster University, Hamilton, ON Canada; 2 Department of Pharmacognosy, Alexandria University, Alexandria, Egypt; 3 Chanchlani Research Centre, McMaster University, Hamilton, Canada; 4 Department of Chemistry and Chemical Biology, McMaster University, Hamilton, ON Canada; 5 Department of Health Research Methods, Evidence, and Impact, Faculty of Health Sciences, McMaster University, Hamilton, ON Canada; 6 Population Health Research Institute, Hamilton Health Sciences, Hamilton, ON Canada; 7 Centre for Metabolism, Obesity and Diabetes Research, McMaster University, Hamilton, ON Canada; 8 Department of Pediatrics, McMaster University, Hamilton, ON Canada; 9 Department of Pediatrics and Child Health, Children’s Hospital Research Institute of Manitoba, University of Manitoba, Winnipeg, MB Canada; 10 Department of Pediatrics, Hospital for Sick Children, Toronto, ON Canada; 11 Department of Pediatrics, University of Alberta, Edmonton, AB Canada; 12 Department of Pediatrics, BC Children’s Hospital, University of British Columbia, Vancouver, BC Canada

**Keywords:** Childhood overweight, Childhood adiposity, Waist circumference, Serum metabolomics, Sex differences, Aromatic amino acids

## Abstract

**Background:**

Childhood obesity is a global health concern and can lead to lifetime cardiometabolic disease. New advances in metabolomics can provide biochemical insights into the early development of obesity, so we aimed to characterize serum metabolites associated with overweight and adiposity in early childhood and to stratify associations by sex.

**Methods:**

Nontargeted metabolite profiling was conducted in the Canadian CHILD birth cohort (discovery cohort) at age 5 years (*n* = 900) by multisegment injection-capillary electrophoresis-mass spectrometry. Clinical outcome was defined using novel combined measures of overweight (WHO-standardized body mass index ≥ 85th percentile) and/or adiposity (waist circumference ≥ 90th percentile). Associations between circulating metabolites and child overweight/adiposity (binary and continuous outcomes) were determined by multivariable linear and logistic regression, adjusting for covariates and false discovery rate, and by subsequent sex-stratified analysis. Replication was assessed in an independent replication cohort called FAMILY at age 5 years (*n* = 456).

**Results:**

In the discovery cohort, each standard deviation (SD) increment of branched-chain and aromatic amino acids, glutamic acid, threonine, and oxoproline was associated with 20–28% increased odds of overweight/adiposity, whereas each SD increment of the glutamine/glutamic acid ratio was associated with 20% decreased odds. All associations were significant in females but not in males in sex-stratified analyses, except for oxoproline that was not significant in either subgroup. Similar outcomes were confirmed in the replication cohort, where associations of aromatic amino acids, leucine, glutamic acid, and the glutamine/glutamic acid ratio with childhood overweight/adiposity were independently replicated.

**Conclusions:**

Our findings show the utility of combining measures of both overweight and adiposity in young children. Childhood overweight/adiposity at age 5 years has a specific serum metabolic phenotype, with the profile being more prominent in females compared to males.

**Supplementary Information:**

The online version contains supplementary material available at 10.1186/s12916-023-02886-8.

## Background

Obesity has become a hallmark of modern childhood with over 340 million children and adolescents with overweight or obesity worldwide [1, 2]. Its growing prevalence in children, including a disproportionate rise during the COVID-19 pandemic, likelihood of leading to lifetime obesity, and association with cardiometabolic risk factors are major concerns [3–5].

Various methods are available for assessing obesity including body mass index (BMI), waist circumference, waist to height ratio, and body fat percentage estimated from sum of skin thickness or more quantitative measures (e.g., bioelectrical impedance analysis) [6]. While BMI standardized for age and sex remains the most commonly used metric of obesity in children, other adiposity measures of central body fat distribution such as waist circumference, and visceral fat are less investigated in children [7, 8]. In all cases, metabolite derived biomarkers hold promise in improved prediction of future cardiometabolic disease than weight or other traditional risk factors alone even at early life stages [9, 10].

State-of-the-art mass spectrometry (MS) or nuclear magnetic resonance (NMR) are major instrumental platforms used in metabolomics analysis of complex biological sample [11, 12]. As substrates or products of biochemical pathways, metabolites capture the output of upstream processes involving the genome, the transcriptome, and the proteome, as well as downstream changes in lifestyle, diet, and environmental exposures [13, 14].

A systematic review of metabolomic studies of obesity in children (age range of 0.25 to 18 years) in the past 15 years resulted in a total of 41 articles and reported an emerging metabolic profile of childhood obesity most consistent for branched-chain and aromatic amino acids [15]. However, the literature in young children (specifically < 6 years) is limited. Given that insulin resistance is usually only evident around puberty, as shown in the longitudinal study of the development of insulin resistance from age 5 to 16 years [16], studying overweight/adiposity during early childhood, e.g., at age 5 years, offers a novel window to investigate how early subclinical metabolic perturbations develop. Such investigations are valuable especially that childhood adiposity is a predictor of cardiometabolic health in adulthood. Likewise, no studies have researched sex-specific differences in early childhood despite the opportunity this could offer in understanding early pathophysiology and optimizing nutrition or growth rate intervention windows based on sex. We hypothesized that there would be sex-related differences in metabolic-adiposity profiles prior to and apart from puberty-related differences.

In the present study, we sought to (i) characterize serum metabolites associated with overweight/adiposity measures in 900 children at 5 years of age, (ii) investigate early sex-dependent differences, and (iii) validate our results in an independent cohort of 456 children of the same age.

## Methods

### Study design

This study was embedded in the CHILD Cohort Study (discovery cohort), a general population-based Canadian birth cohort that recruited pregnant women from Toronto, Manitoba, Edmonton, and Vancouver from 2009 to 2012 [17]. Of 3278 infants enrolled in CHILD, 3098 completed a follow-up 5-year visit, 1770 had serum specimens available, and of these 900 children had complete metabolomics data available (consort diagram Additional File 1: Figure S1). Replication of metabolite biomarker candidates was tested among 456 children age 5 years with complete metabolomics data, as previously published [18], from the Family Atherosclerosis Monitoring In earLY life (FAMILY) prospective birth cohort study (replication cohort) [19], analyzed by the same validated instrumental platform as used in CHILD [20].

Non-fasting blood samples for CHILD and fasting blood samples for FAMILY were collected and serum was fractionated within 24 h from collection according to standard protocols, stored at – 80 °C and shipped on dry ice [6]. Child anthropometric measures included weight, height, waist circumference, and skin fold thickness (the sum of subscapular and triceps skinfolds). BMI was calculated as weight in kilograms divided by height in meters squared, and standardized BMI-for-age *z*-scores were derived based on the World Health Organization (WHO) child growth standards [6]. Age- and sex-standardized waist circumference z-scores were derived based on percentile curves created for 18,745 children aged 2.0–10.9 years of the European IDEFICS (Identification and prevention of Dietary-and lifestyle-induced health EFfects in children and infantS) cohort [21].

**Fig. 1:**
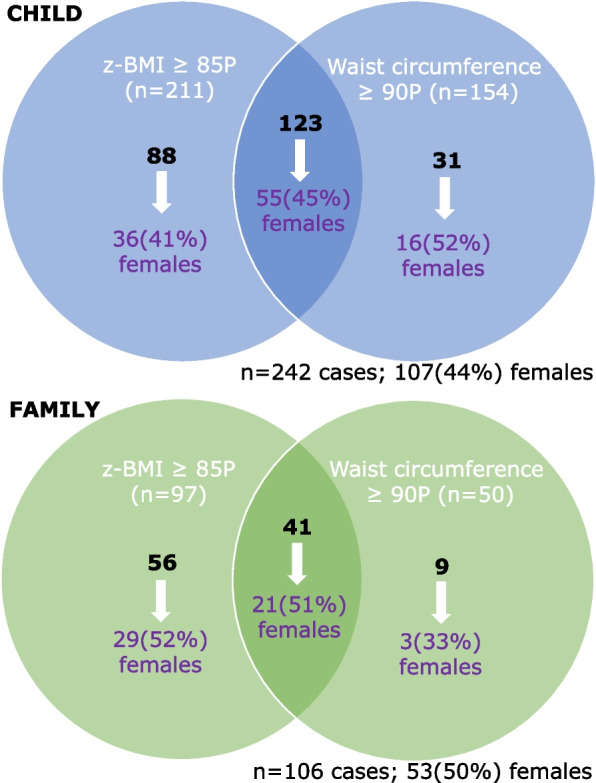
Overweight/adiposity cases at age 5 years in CHILD and FAMILY birth cohorts

Children were classified as overweight if their z-BMI ≥ 85th percentile (BMI-for-age greater than 1 standard deviation above the WHO growth reference median) or high adiposity if their waist circumference was ≥ 90th percentile (cohort- and sex-specific) [22, 23]. Two hundred forty-two children in CHILD and 106 children in FAMILY met these criteria and were classified as cases (Fig. 1). If neither of the criteria was met, children were classified as non-cases (658 children in CHILD and 350 children in FAMILY).

Data on covariates were obtained from standardized questionnaires, including night sleep (hours/day), screen time (< 2 h/day cutoff as per Canadian guidelines recommendations) [24], social disadvantage index (composed of household income, marital status and maternal employment) [25], maternal education (years), breastfeeding status at 1 year (whether or not the child was breastfed up to the first year of life, irrespective of breastfeeding exclusivity), diet quality score [26, 27], and physical activity level (average hours per week spent in moderately vigorous physical activity participating in organized and unorganized sports and activities, outside of school hours) [18].

### Serum metabolomics analysis

A high-throughput platform based on multisegment injection-capillary electrophoresis-mass spectrometry (MSI-CE-MS) was used for the analysis of polar ionic metabolites in serum filtrate samples as described in detail elsewhere [28]. Briefly, an Agilent 6230 time-of-flight MS with a coaxial sheath liquid Jetstream electrospray ion source equipped to an Agilent G7100A CE was used for data acquisition. This multiplexed separation platform takes advantage of a serial sample injection format of 13 samples within a single CE run including a pooled quality control (QC) sample prepared by combining equal aliquots of serum filtrate samples from all study participants for assessment of technical precision [29, 30]. An iterative data workflow based on temporal signal pattern recognition with serial sample injections was used to reject spurious signals, redundant peaks, and background ions when performing nontargeted metabolomics to report authenticated serum metabolites [20, 30].

Fifty-one serum metabolites were consistently measured in over 75% of the samples and satisfied QC criteria of a variance under 30% when using MSI-CE-MS under 2 configurations with positive and negative-ion mode detection. Forty-six metabolites were unambiguously identified (level 1) and subsequently quantified using a calibration curve, where ion responses were normalized to an internal standard (Additional File 2: Table S1). Four unknown serum metabolites were annotated based on their characteristic accurate mass and relative migration time under positive or negative ion mode. Metabolite combinations and ratios frequently used in the literature were derived for branched-chain amino acids (BCAAs) as the sum of leucine, isoleucine, and valine, aromatic amino acids (AAAs) as the sum of tyrosine and phenylalanine, and the glutamine/glutamic acid ratio.

Of note, fasting status was the only differing collection parameter between the discovery and validation cohorts, while type of specimen, collection and storage parameters, sample preprocessing, and metabolomics analysis were identical between cohorts.

### Statistical analysis

Descriptive statistics for anthropometrics, age, blood pressure, and lifestyle factors were presented as mean and standard deviation overall (*n* = 900) and stratified by cases (*n* = 242) and non-cases (*n* = 658), for children 5 years of age. Categorical variables were presented as counts and percentages. All metabolite concentrations (or relative peak area if standard was unavailable) were natural-log transformed before analyses to obtain approximately normal distributions. They were subsequently scaled to standard deviation (SD) units for each cohort. Significant metabolite associations with pediatric overweight/adiposity at age 5 years were tested separately for each metabolite using multivariable logistic regression. Similarly, sex-dependent differences were tested in multivariable logistics regression models stratified by child sex. Based on the literature and prior knowledge, a parsimonious set of covariates that associate with childhood obesity were initially selected as listed under study design. All variables significant by association with our categorical or continuous outcomes in a simple univariate regression (*α* ≤ 0.10) were entered into backward elimination selection procedures, whereby the sum of BCAAs was included in the global model as a representative of metabolite exposures because it shows the strongest association to childhood obesity in the literature [15]. As a result, our final multivariable models adjusted for maternal education, child night sleep at age 5 years, breastfeeding status at 1 year, in addition to child age and sex. The same analysis was conducted using linear regression models with the continuous measure of z-BMI, and adiposity (i.e., waist circumference, and sum of skinfolds) as the outcome variables. Statistical significance for the multivariable regression analyses was set at *p* < 0.05 and a Benjamini–Hochberg false discovery rate (FDR) of 0.1 was considered to adjust for multiple testing.

Among the 900 study participants in CHILD, all covariates were available for 696 children (77.3%). To test consistency of results, missing values of covariates, assumed to be missing at random, were multiple imputed (*n* = 20 imputations) using the MICE (Multivariate Imputation by Chained Equations) R package that uses a conditional multiple imputation approach. Estimates were pooled from all 20 imputed datasets. Metabolites associated with the overweight/adiposity combined dichotomous outcome were then analyzed in the FAMILY validation cohort with full adjustment and subsequent sex-stratified analysis. Among the 456 study participants in FAMILY, all covariates were available for 420 children (92%). All analyses, tables, and graphs were completed in R (v3.6.3; R Foundation).

## Results

### Participants’ characteristics

The discovery cohort included 900 children from the CHILD prospective birth cohort and demographic characteristics are summarized in Table 1. Briefly, the mean age of participants was 5.03 years; and 45% were girls. One hundred twenty-three children met both z-BMI and waist circumference criteria for case selection, while 88 and 31 children were above either the z-BMI or the waist circumference thresholds, respectively (Fig. 1). The mean z-BMI score was 1.69 and 0.001, and the mean waist circumference was 58.91 cm and 50.95 cm, in children classified as cases versus in non-cases respectively. For the replication cohort, metabolite biomarker candidates were measured in 456 children from FAMILY with a mean age of 5.15 years and 50% were girls (Additional File 3: Table S2, Fig. 1).

**Table 1:** Baseline characteristics of the CHILD study population

**Characteristic**	**Cases** **(*n* = 242)**	**Non-cases** **(*n* = 658)**	**All children** **(*n* = 900)**
**Age (years)**	5.02 (0.11)	5.04 (0.15)	5.03 (0.14)
**Sex (female)**	107 (44%)	301 (46%)	408 (45%)
**Daily night sleep (hours)**	10.91 (0.72)	11 (0.75)	10.98 (0.74)
**Physical activity (hours/week)**	11.06 (9.28)	10.55 (8.73)	10.67 (8.86)
**Diet quality score**	0.21 (1.75)	0.31 (2.1)	0.28 (2.02)
**Maternal education (years)**	15.64 (3.24)	16.94 (3.15)	16.63 (3.22)
**Social disadvantage index**			
** Low 0–1**	101(71.0%)	351 (76.0%)	452 (75.0%)
** Moderate 2–3**	39 (27.0%)	99 (21.0%)	138 (23.0%)
** High 4–5**	3 (2.0%)	11 (3.0%)	14 (2.0%)
**Screen time exposure**			
** Low exposure (< 2 h)**	112 (61%)	368 (67%)	480 (78%)
** High exposure (≥ 2 h)**	73 (39%)	181 (33%)	254 (22%)
**Systolic blood pressure (mm Hg)**	107.59 (10.04)	100.58 (9.27)	102.25 (9.91)
**Diastolic blood pressure (mm Hg)**	60.88 (7.04)	57.94 (5.8)	58.64 (6.24)
**BMI (kg/m**^**2**^**)**	18.0 (1.94)	15.3 (0.90)	16.02 (1.77)
**BMI-for-age *z*-score (WHO)**	1.69 (0.99)	0.001 (0.67)	0.45 (1.08)
**Waist circumference (cm)**	58.91 (4.44)	50.95 (3.55)	52.85 (5.08)
**Waist circumference *z*-score**	2.17 (0.99)	− 0.19 (1.19)	0.45 (1.54)
**Waist circumference-to-height**	0.52 (0.04)	0.46 (0.03)	0.48 (0.04)
**Sum of skinfolds (mm)**	20.62 (5.86)	15.24 (3.63)	16.53 (4.84)

Values are presented as mean (SD) or n (%). Diet quality score: sum of daily servings of “healthy” foods less the sum of daily servings of “unhealthy” foods

### Metabolite profiling of childhood overweight/adiposity at age 5 years

#### Comparison of serum metabolite profiles in cases and non-cases

We examined each serum metabolite for association with the overweight/adiposity case-non-case status. Eleven metabolite markers were statistically different between cases and non-cases in the unadjusted, as well as the adjusted multivariable model, and passed FDR adjustment as shown in Table 2. The top metabolite markers included BCAAs and AAAs. For the three BCAAs, leucine, valine, and isoleucine, their sum, and the two AAAs, tyrosine, phenylalanine, and their sum, each SD increment in log serum biomarker was associated with 20–28% increased odds of child overweight/adiposity, whereas the glutamine/glutamic acid ratio was associated with 20% decreased odds. Glutamic acid, threonine, and oxoproline were also positively associated with overweight/adiposity at age 5 years. Results were consistent among other intermediate adjusted models adjusting for age and sex only, and age, sex, and maternal education (proxy for socioeconomic status), as well as the multiple imputed models (Additional File 4: Table S3).

**Table 2:** Serum metabolites associated with child overweight/adiposity at age 5 years in CHILD^a^

**Metabolite**	**OR**	**95% CI**	***p*-**	**FDR**
**Leucine**	1.28	(1.07–1.54)	0.007	0.009
**BCAAs**	1.27	(1.06–1.52)	0.009	0.018
**Valine**	1.26	(1.06–1.51)	0.01	0.027
**AAAs**	1.23	(1.03–1.48)	0.024	0.036
**Isoleucine**	1.23	(1.03–1.47)	0.025	0.045
**Glutamic acid**	1.28	(1.04–1.61)	0.025	0.054
**Threonine**	1.23	(1.02–1.47)	0.029	0.063
**Glutamine/glutamic acid**	0.81	(0.66–0.98)	0.039	0.072
**Phenylalanine**	1.22	(1.01–1.47)	0.042	0.082
**Tyrosine**	1.19	(1.00–1.42)	0.045	0.09
**Oxoproline**	1.22	(1.01–1.48)	0.045	0.1

*BCAAs* branched-chain amino acids, *AAAs* aromatic amino acids, *OR* odds ratio, *95*% *CI* confidence intervals, *p- p*-value for statistical significance, *FDR* false discovery rate *d* = 0.1; when *p*- is smaller than this value, association passes multiple hypothesis testing

^a^Multivariable logistic regression adjusting for maternal education, sleep time, breastfeeding status at 1 year, sex, and age [204 (23%) had missing values on at least one covariate; complete cases analysis *n* = 696: 173 cases and 523 non-cases]

#### Investigation of early sex-dependent differences in metabolite markers

**Fig. 2:**
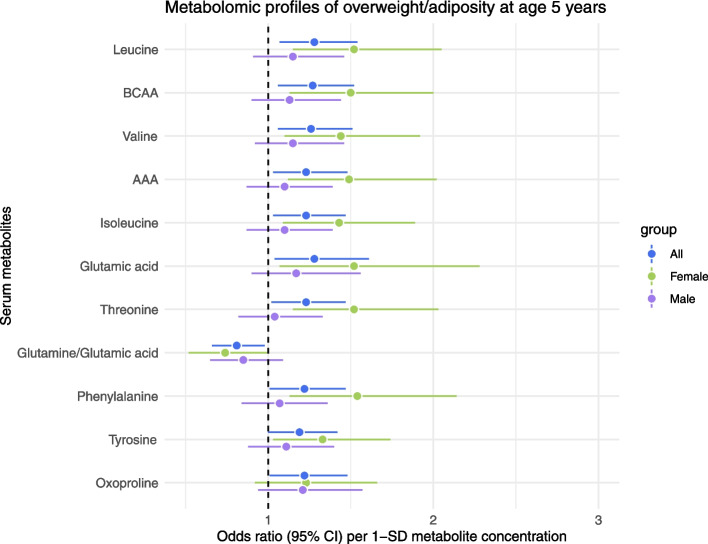
Serum metabolite associations with overweight/adiposity in the CHILD birth cohort categorized by sex (female and male) compared to non-stratified analysis (all) depicting OR and 95% CI. OR are per 1-SD log-transformed metabolite concentrations and adjusted for child’s age and sleep time at 5 years, breastfeeding status at 1 year, and maternal education

Next, sex-stratified analysis was conducted for the resulting 11 serum metabolite markers listed in Table 2. Figure 2 illustrates the odds ratio (OR) and 95% confidence intervals (CI) of the metabolite associations with overweight/adiposity categorized by sex. Separate multivariable logistic regression models for 387 males and 309 females were adjusted for age, maternal education, sleep time, and breastfeeding status at 1 year in 97 (25%) cases and 290 (75%) non-cases and in 76 (25%) cases and 233 (75%) non-cases, respectively. Almost all metabolite associations (except for oxoproline) were only significant in females (49–64% increased odds for top metabolite panel) indicating nearly twofold greater estimates of overweight/adiposity among females for most metabolites, while the corresponding ORs for males were not significant.

#### Comparison of performance of different body composition metrics

**Fig. 3:**
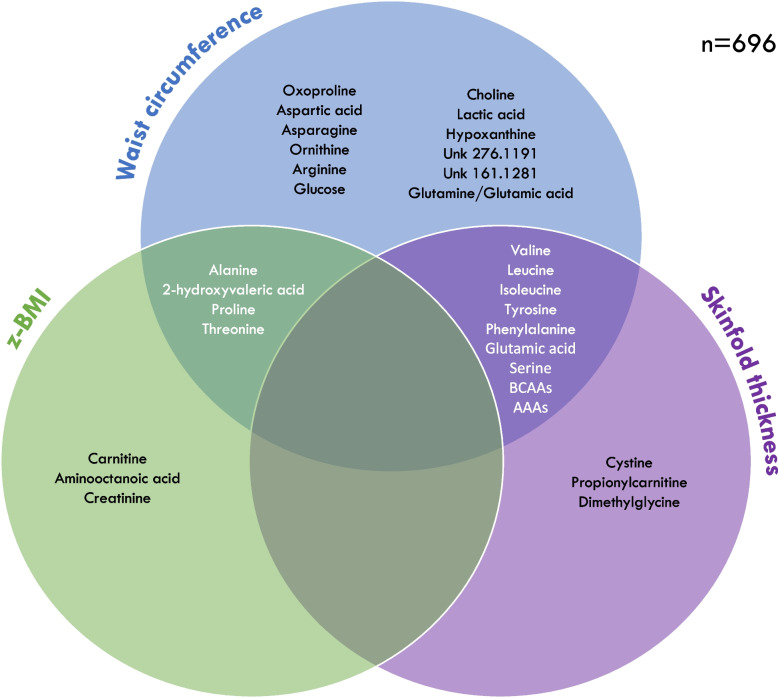
Venn diagram of the metabolites significantly associated with z-BMI, waist circumference and sum of skinfold and their overlap in 696 children in CHILD at age 5 years

Multivariable linear regression models were used to investigate serum metabolite associations with three continuous anthropometric measures, namely z-BMI, waist circumference/z-waist circumference, and sum of skinfold (*n* = 696). Figure 3 shows a Venn diagram of the number and names of serum metabolites significantly associated with each measure and their overlap while Additional File 5: Table S4, Additional File 6: Table S5, and Additional File 7: Table S6 present the regression results for z-BMI, waist circumference, and sum of skinfold, respectively. Using the results of the logistic regression analysis as reference for the 11 significant metabolites from the logistic regression analysis, waist circumference as a continuous outcome performed best in terms of number, magnitude, and strength of metabolite associations, followed by sum of skin folds, then z-BMI. Standardized z-waist circumference depicted the same results (data not shown). None of the 11 serum metabolites markers was common across all three anthropometric measures. BCAAs, AAAs, and glutamic acid associations were broadly similar across waist circumference and sum of skin folds, although the strength of specific associations showed some variation, and were not significantly associated with z-BMI. Threonine was significantly associated with both z-BMI and waist circumference, while oxoproline and the glutamine/glutamic acid ratio with waist circumference only.

#### Validation of metabolomics findings in FAMILY at age 5 years

**Fig. 4:**
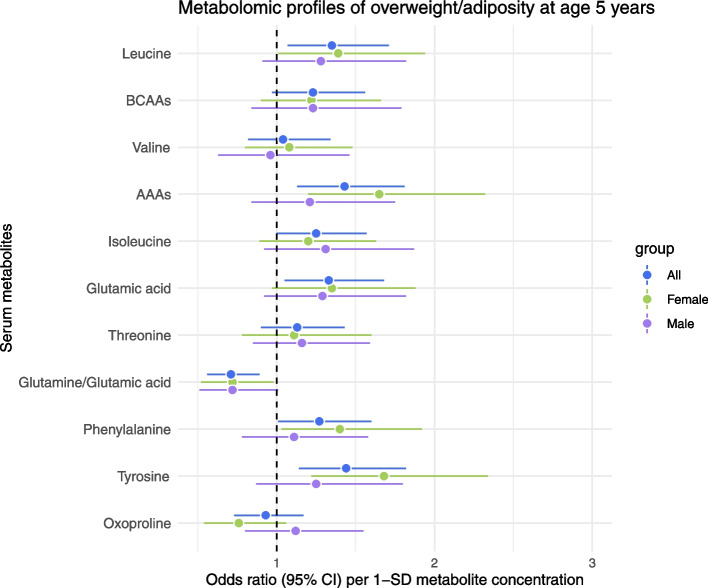
Metabolite associations with overweight/adiposity in the FAMILY replication cohort categorized by sex (female and male) compared to non-stratified analysis (all) depicting OR and 95% CI. OR are per 1-SD log-transformed metabolite concentrations and adjusted for child’s age, sleep time, breastfeeding status at 1 year, and maternal education

The 11 serum biomarker candidates identified from the logistic regression analysis of overweight/adiposity in the CHILD discovery cohort were then tested in the FAMILY replication cohort. Importantly, serum samples in FAMILY were collected when participants were fasting as opposed to the non-fasting serum samples in the discovery cohort. Six of the 11 metabolite measures were significantly associated with overweight/adiposity in FAMILY as shown in Fig. 4 and Additional File 8: Table S7. Higher tyrosine, phenylalanine, AAAs, leucine, and glutamic acid, and lower glutamine/glutamic acid ratio were associated with overweight/adiposity. The same sex-specific trend observed in CHILD was also observed in FAMILY, where the six replicated metabolites, were only significant in females (*n* = 213; 50 cases) but not in males (*n* = 207; 46 cases) in sex-stratified analyses.

## Discussion

We identified a robust metabolite phenotype that was associated with overweight and adiposity in young children. This panel is comprised of BCAAs and AAAs, glutamic acid, the glutamine/glutamic acid ratio, and threonine and was more evident in female children in a well-characterized cohort of 900 children and a replication cohort of 456 children at age 5 years. More direct measures of adiposity/fat distribution including waist circumference and sum of skinfold thickness yielded more significant associations than did z-BMI.

Our metabolomic phenotype findings are generally in agreement with prior obesity metabolome investigations in older children (7.7–11.1 years of age) despite the non-fasting status in our discovery cohort [31, 32]. Our study suggests that BCAAs in children do not primarily associate with postprandial interval [33] because the serum samples collected in CHILD were non-fasting—a finding that has also been made in adults [34, 35]. Our observation, that BCAAs concentration is associated with overweight and adiposity at 5 years of age, is most consistent with the literature as reported by at least 10 prior studies in children of varying age [15]. In studies with contrasting findings, BCAAs concentrations were lower in adolescents with obesity and type 2 diabetes compared with adolescents with both normal weight or with obesity [36] and were inversely associated with insulin resistance in a longitudinal follow-up of children (age 5–16) [16]. Of note, in our FAMILY replication cohort, we have observed significant negative associations of BCAAs with fasting serum glucose (data not shown). Furthermore, we have previously identified a distinct metabolomic phenotype associated with an aggregate metabolic syndrome risk score in the FAMILY cohort that did not include BCAAs [18]. Congruent with our current analysis, this metabolic syndrome signature included the aromatic amino acid tyrosine, threonine, alanine, and glutamine/glutamic acid ratio. These could be promising biomarkers of cardiometabolic disease risk in young children reflecting an aggregate of risk factors that includes but is not limited to excessive abdominal adiposity [18, 37].

Most studies in the literature have used z-BMI to classify childhood obesity, while other adiposity measures of central body fat distribution such as waist circumference, or skin fold thickness are infrequently measured or investigated among children. Using linear regression analysis, we have shown that metabolic differences can arise depending on whether an obesity or adiposity measure is used, with a greater number of serum metabolites being identified in relationship to adiposity rather than overweight/obesity. In accordance with research showing the independent and additive value of waist circumference metric in predicting morbidity [38], the approach we adopted by combining both z-BMI and waist circumference may serve as a useful approach for other researchers and future investigations. Few prior studies have investigated the variation of the overweight/adiposity metabolome in children by sex. Most studies included older children and youth where puberty changes become a major confounder [9, 10, 39–42]. In our sex-stratified analysis, we observed that the metabolic profile associated with overweight and adiposity at age 5 years was mainly driven by the effects in girls, while boys showed mostly weaker and non-significant associations (albeit in the same direction). Saner et al. investigated sex and puberty-related metabolic differences related to adiposity in youth [mean age 11.9 (3.1 SD)] and found that tyrosine and phenylalanine were positively associated with z-BMI in girls only, consistent with our results, whereas in boys significant associations with leucine and isoleucine were seen post-puberty [9]. These sex-specific differences could be related to girls maturing earlier than boys but unlikely to be a direct result of sexual hormone maturation because adrenarche (even premature adrenarche) occurs at an older age than that of our cohort. Another hypothesis to explain our sex-related findings may be linked to differences in mitochondrial plasticity between boys and girls—a process of mitochondrial adaptation to normalize/correct metabolic impairments [36]. Further sex-specific analyses are necessary, ideally within longitudinal studies, to better understand the underpinning mechanisms and to test these hypotheses of sex- and puberty-related metabolomic associations with obesity and cardiometabolic disease.

Our study has several strengths which include its large sample size (1345 children) and high quality as defined by Handakas et al. [15]. In this systematic review of 41 metabolomic studies of childhood obesity, 66% of studies had a sample size < 200, and few were rated as high quality [15]. Furthermore, our study addresses the disparity in research of sex-based differences in metabolic changes in early childhood. Moreover, we included a validation of our findings in an independent cohort of children of the same age using the same validated metabolomics platform with rigorous quality control. Several limitations should also be considered. Serum samples were non-fasting in CHILD. Metabolome coverage was limited to polar ionic metabolites and did not include important circulating lipids, such as fatty acids and phospholipids [43, 44]; thus, complementing our analysis with other orthogonal platforms would be valuable. Finally, this is a cross-sectional evaluation and it is yet unknown if the overweight/adiposity metabolomic profiles are linked to future health outcomes.

## Conclusions

Using metabolomics profiling of 1345 children, we have identified that BCAAs, AAAs, glutamic acid, and the glutamine/glutamic acid ratio are associated with overweight and adiposity in early childhood at age 5 years, and this relationship is more evident in girls than boys at age 5 years. The use of central adiposity measures, e.g., waist circumference, was optimal in defining this metabolomic pattern. Overall, our work underscores the value of high-throughput metabolomics in understanding disease pathways and the need for future sex-specific investigations to design future interventions to mitigate the obesity pandemic as early in life as possible.

## Supplementary Information

**Additional file 1: Figure S1.** Flowchart for overweight/adiposity case selection in CHILD.

**Additional file 2: TableS1.** Serum metabolites characteristics.

**Additional file 3: Table S2.** FAMILY baseline characteristics.

**Additional file 4: Table S3.** Multiple imputation analysis.

**Additional file 5: Table S4.** Metabolomics of waist circumference.

**Additional file 6: Table S5.** Metabolomics of body mass index.

**Additional file 7: Table S6.** Metabolomics of sum of skinfolds.

**Additional file 8: Table S7.** Results of the FAMILY validation cohort.

## Data Availability

The datasets used and/or analyzed during the current study are available from the corresponding author on reasonable request.

## Abbreviations

BMI: Body mass index
MS: Mass spectrometry
NMR: Nuclear magnetic resonance
FAMILY: Family Atherosclerosis Monitoring In earLY life
WHO: World Health Organization
IDEFICS: Identification and prevention of Dietary-and lifestyle-induced health EFfects in children and infants cohort
MSI-CE-MS: Multisegment injection-capillary electrophoresis-mass spectrometry
QC: Quality control
SD: Standard deviation
BCAAs: Branched-chain amino acids
AAAs: Aromatic amino acids
FDR: False discovery rate
MICE: Multivariate imputation by chained equations
OR: Odds ratio
CI: Confidence intervals

## References

[1] Lobstein CT, Brinsden H. Atlas of Childhood Obesity. World Obesity Federation Charles Darwin 2, 107 Gray’s Inn Road, London, WC1 X8TZ; 2019. p. 212. https://www.worldobesity.org/membersarea/global-atlas-on-childhood-obesity.

[2] Health Organization. Obesity and Overweight. Available online: https://www.who.int/news-room/fact-sheets/detail/obesity-and-overweight. Accessed on 1 Dec 2021.

[3] Hauerslev M, Narang T, Gray N, Samuels TA, Bhutta ZA (2022). Childhood obesity on the rise during COVID-19: a request for global leaders to change the trajectory. Obesity.

[4] Abarca-Gómez L, Abdeen ZA, Hamid ZA (2017). Worldwide trends in body-mass index, underweight, overweight, and obesity from 1975 to 2016: a pooled analysis of 2416 population-based measurement studies in 128·9 million children, adolescents, and adults. Lancet.

[5] Jacobs DR, Woo JG, Sinaiko AR (2022). Childhood cardiovascular risk factors and adult cardiovascular events. N Engl J Med.

[6] Teo KK, Rafiq T, Anand SS (2019). Associations of cardiometabolic outcomes with indices of obesity in children aged 5 years and younger. PLoS One.

[7] Weiss R, Tamborlane WV, Allen K, Sherwin RS. Obesity and the metabolic syndrome in children and adolescents. N Engl J Med. 2004;350(23):2362-74. 10.1056/NEJMoa031049 15175438

[8] Medehouenou TCM, Roy C, Tremblay PY (2021). Metabolic features of adiposity and glucose homoeostasis among school-aged inuit children from Nunavik (Northern Quebec, Canada). Int J Circumpolar Health.

[9] Saner C, Harcourt BE, Pandey A (2019). Sex and puberty-related differences in metabolomic profiles associated with adiposity measures in youth with obesity. Metabolomics.

[10] Perng W, Rifas-Shiman SL, Hivert MF, Chavarro JE, Oken E (2018). Branched chain amino acids, androgen hormones, and metabolic risk across early adolescence: a prospective study in Project Viva: BCAA, androgens, and metabolic risk in adolescence. Obesity.

[11] Wang TJ, Larson MG, Vasan RS (2011). Metabolite profiles and the risk of developing diabetes. Nat Med.

[12] Newgard CB (2017). Metabolomics and metabolic diseases: where do we stand?. Cell Metab.

[13] Dunn WB, Broadhurst DI, Atherton HJ, Goodacre R, Griffin JL (2011). Systems level studies of mammalian metabolomes: the roles of mass spectrometry and nuclear magnetic resonance spectroscopy. Chem Soc Rev.

[14] Wishart DS (2019). Metabolomics for investigating physiological and pathophysiological processes. Physiol Rev.

[15] Handakas E, Lau CH, Alfano R, et al. A systematic review of metabolomic studies of childhood obesity: State of the evidence for metabolic determinants and consequences. Obes Rev. 2022;23(S1). 10.1111/obr.13384 10.1111/obr.13384 34797026

[16] Hosking J, Pinkney J, Jeffery A (2019). Insulin Resistance during normal child growth and development is associated with a distinct blood metabolic phenotype (Earlybird 72). Pediatr Diabetes.

[17] Subbarao P, Anand SS, Becker AB (2015). The Canadian Healthy Infant Longitudinal Development (CHILD) Study: examining developmental origins of allergy and asthma: Table 1. Thorax.

[18] Azab SM, de Souza RJ, Lamri A (2021). Metabolite profiles and the risk of metabolic syndrome in early childhood: a case-control study. BMC Med.

[19] Morrison KM, Atkinson SA, Yusuf S (2009). The Family Atherosclerosis Monitoring In earLY life (FAMILY) study. Am Heart J.

[20] Shanmuganathan M, Kroezen Z, Gill B (2021). The maternal serum metabolome by multisegment injection-capillary electrophoresis-mass spectrometry: a high-throughput platform and standardized data workflow for large-scale epidemiological studies. Nat Protoc.

[21] Ahrens W, Pigeot I, Pohlabeln H (2014). Prevalence of overweight and obesity in European children below the age of 10. Int J Obes.

[22] Yusuf S, Hawken S, Ôunpuu S (2004). Effect of potentially modifiable risk factors associated with myocardial infarction in 52 countries (the INTERHEART study): case-control study. Lancet.

[23] Taxová Braunerová R, Kunešová M, Heinen MM, et al. Waist circumference and waist-to-height ratio in 7-year-old children—WHO Childhood Obesity Surveillance Initiative. Obes Rev. 2021;22(S6). 10.1111/obr.13208 10.1111/obr.13208 34402567

[24] Colley RC, Garriguet D, Adamo KB (2013). Physical activity and sedentary behavior during the early years in Canada: a cross-sectional study. Int J Behav Nutr Phys Act.

[25] Anand SS, Razak F, Davis A (2006). Social disadvantage and cardiovascular disease: development of an index and analysis of age, sex, and ethnicity effects. Int J Epidemiol.

[26] de Souza RJ, Zulyniak MA (2016). Harmonization of food-frequency questionnaires and dietary pattern analysis in 4 ethnically diverse birth cohorts. J Nutr.

[27] de Souza RJ, Shanmuganathan M, Lamri A (2020). Maternal diet and the serum metabolome in pregnancy: robust dietary biomarkers generalizable to a multiethnic birth cohort. Curr Dev Nutr.

[28] Kuehnbaum NL, Kormendi A, Britz-McKibbin P (2013). Multisegment injection-capillary electrophoresis-mass spectrometry: a high-throughput platform for metabolomics with high data fidelity. Anal Chem.

[29] Azab SM, Zamzam A, Syed MH, Abdin R, Qadura M, Britz-McKibbin P (2020). Serum metabolic signatures of chronic limb-threatening ischemia in patients with peripheral artery disease. J Clin Med.

[30] Saoi M, Li A, McGlory C (2019). Metabolic perturbations from step reduction in older persons at risk for sarcopenia: plasma biomarkers of abrupt changes in physical activity. Metabolites.

[31] Perng W, Gillman MW, Fleisch AF (2014). Metabolomic profiles and childhood obesity: metabolomic profiles and childhood obesity. Obesity.

[32] Butte NF, Liu Y, Zakeri IF (2015). Global metabolomic profiling targeting childhood obesity in the Hispanic population. Am J Clin Nutr.

[33] Lau CHE, Siskos AP, Maitre L (2018). Determinants of the urinary and serum metabolome in children from six European populations. BMC Med.

[34] Tobias DK, Mora S, Verma S, Lawler PR (2018). Altered branched chain amino acid metabolism: toward a unifying cardiometabolic hypothesis. Curr Opin Cardiol.

[35] Tobias DK, Mora S, Verma S, Billia F, Buring JE, Lawler PR. Fasting status and metabolic health in relation to plasma branched chain amino acid concentrations in women. Metabolism. 2020:154391. 10.1016/j.metabol.2020.154391 10.1016/j.metabol.2020.154391 PMC7985990 33069808

[36] Mihalik SJ, Michaliszyn SF, de las Heras J (2012). Metabolomic profiling of fatty acid and amino acid metabolism in youth with obesity and type 2 diabetes. Diabetes Care.

[37] Hellmuth C, Kirchberg FF, Lass N (2016). Tyrosine is associated with insulin resistance in longitudinal metabolomic profiling of obese children. J Diabetes Res.

[38] Ross R, Neeland IJ, Yamashita S (2020). Waist circumference as a vital sign in clinical practice: a Consensus Statement from the IAS and ICCR Working Group on Visceral Obesity. Nat Rev Endocrinol.

[39] Hirschel J, Vogel M, Baber R (2020). Relation of whole blood amino acid and acylcarnitine metabolome to age, sex, BMI, puberty, and metabolic markers in children and adolescents. Metabolites.

[40] Perng W, Rifas-Shiman SL, Sordillo J, Hivert MF, Oken E (2020). Metabolomic profiles of overweight/obesity phenotypes during adolescence: a cross-sectional study in Project Viva. Obesity.

[41] LaBarre JL, Peterson KE, Kachman MT (2020). Mitochondrial nutrient utilization underlying the association between metabolites and insulin resistance in adolescents. J Clin Endocrinol Metab.

[42] Jeffery SC, Hosking J, Jeffery AN, Murphy MJ, Voss LD, Wilkin TJ (2018). Insulin resistance is higher in prepubertal girls but switches to become higher in boys at age 16: a cohort study (EarlyBird 57): JEFFERY et al. Pediatr Diabetes.

[43] Ly R, Ly N, Sasaki K, et al. Nontargeted serum lipid profiling of nonalcoholic steatohepatitis by multisegment injection–nonaqueous capillary electrophoresis–mass spectrometry: a multiplexed separation platform for resolving ionic lipids. J Proteome Res. 2021. 10.1021/acs.jproteome.1c00682 10.1021/acs.jproteome.1c00682 34676758

[44] Azab S, Ly R, Britz-McKibbin P (2019). Robust method for high-throughput screening of fatty acids by multisegment injection-nonaqueous capillary electrophoresis–mass spectrometry with stringent quality control. Anal Chem.

