# Supplementary figures and images for: Early sex-dependent differences in metabolic profiles of overweight and adiposity in young children: a cross-sectional analysis
Source: BMC Med. 2023 May 9;21:176. doi: 10.1186/s12916-023-02886-8 (PMC10166631; doi:10.1186/s12916-023-02886-8)

**Figure S1. Flow chart for overweight/adiposity case selection in CHILD at age 5 years**

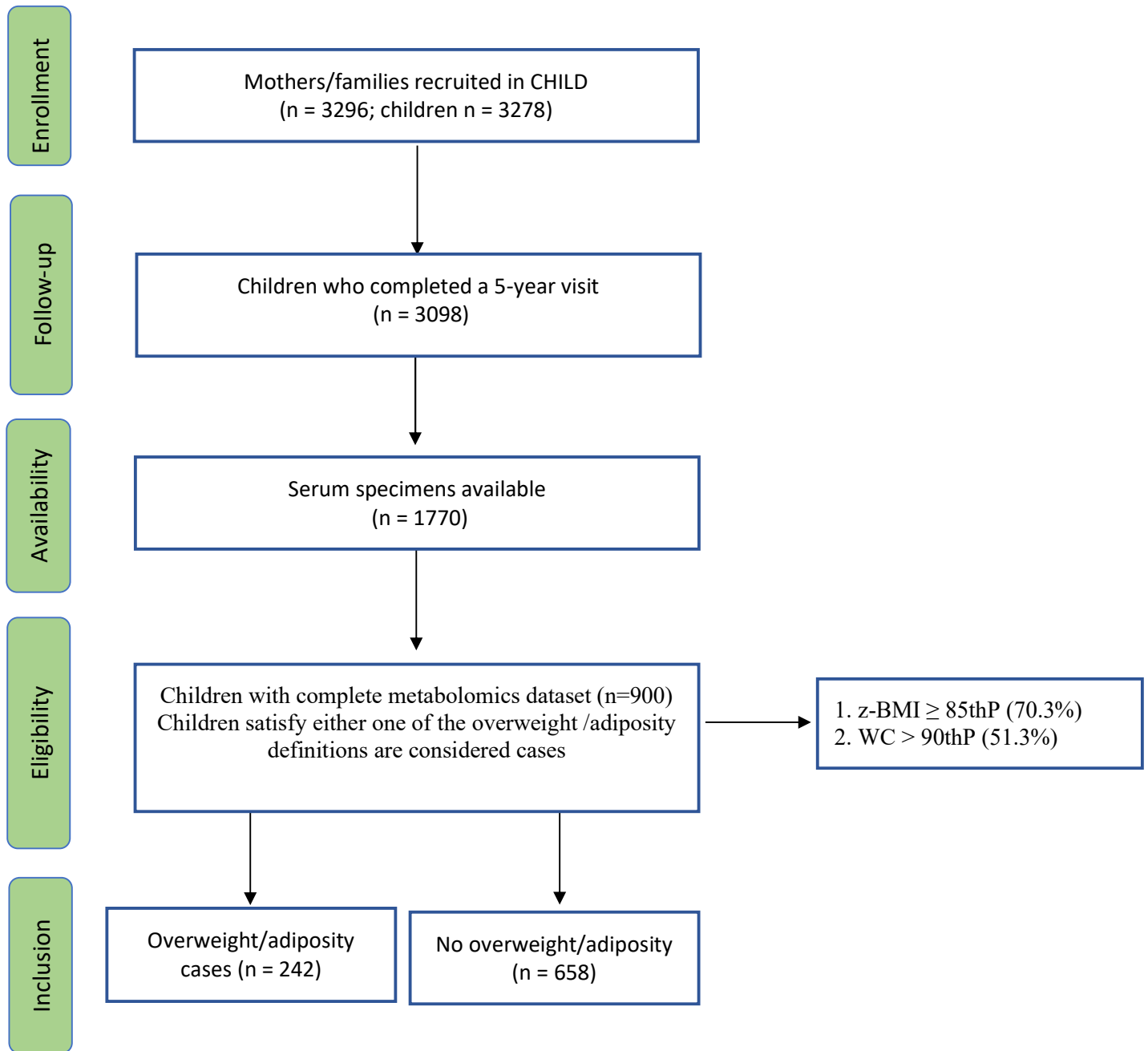

Supplement: Supplementary file 1 — Additional file 1: Figure S1. Flowchart for overweight/adiposity case selection in CHILD. [file 12916_2023_2886_MOESM1_ESM.pdf]
