# Supplementary material for: Early sex-dependent differences in metabolic profiles of overweight and adiposity in young children: a cross-sectional analysis
Source: BMC Med. 2023 May 9;21:176. doi: 10.1186/s12916-023-02886-8 (PMC10166631; doi:10.1186/s12916-023-02886-8)
Supplement: Supplementary file 2 — Additional file 2: TableS1. Serum metabolites characteristics. [file 12916_2023_2886_MOESM2_ESM.docx]

| Table S1. Summary of 51 serum metabolites detected in CHILD five-year old children. | | | | | | |
| --- | --- | --- | --- | --- | --- | --- |
| # | **Metabolite ID** | Molecular Formula | m/z:RMT:Ion mode | Mean concentration  (µM) (SD) | QC (%CV) | Complete Data (%) |
| 1 | **Glycine** | C₂H₅NO₂ | 76.0393:0.55:P | 292.26 (98.20) | 12 | 100 |
| 2 | **Trimethylamine-N-oxide** | C_3_H_9_NO | 76.0766:0.29:P | 5.67 (10.99) | 15.1 | 83.9 |
| 3 | **Alanine** | C_3_H_7_NO_2_ | 90.0556:0.64:P | 435.52 (97.35) | 6.5 | 100 |
| 4 | **Dimethylglycine** | C_4_H_9_NO_2_ | 104.0711:0.71:P | 10.09 (3.24) | 7.7 | 100 |
| 5 | **Choline** | C_5_H_14_NO+ | 104.1075:0.37:P | 48.55 (13.42) | 7.8 | 100 |
| 6 | **Serine** | C_3_H_7_NO_3_ | 106.05:0.77:P | 186.16 (27.20) | 3.3 | 100 |
| 7 | **Creatinine** | C_4_H_7_N_3_O | 114.0662:0.41:P | 30.21 (9.63) | 16.8 | 100 |
| 8 | **Proline** | C₅H₉NO₂ | 116.0705:0.86:P | 231.31 (67.81) | 3 | 100 |
| 9 | **Valine** | C_5_H_11_NO_2_ | 118.0862:0.76:P | 233.82 (54.53) | 5.7 | 100 |
| 10 | **Betaine** | C_5_H_12_NO_2_ | 118.0862:0.92:P | 45.70 (10.77) | 6 | 78 |
| 11 | **Threonine** | C_4_H_9_NO_3_ | 120.0654:0.84:P | 133.48 (28.10) | 3.2 | 100 |
| 12 | **Unknown 1** | C_5_H_8_N_2_O_2_ | 129.066:0.61:P | 0.11 (0.03)* | 16.7 | 100 |
| 13 | **Hydroxyproline** | C_5_H_9_NO_3_ | 132.0655:0.75:P | 3.45 (0.69) | 6.9 | 94.2 |
| 14 | **Creatine** | C_4_H_9_N_3_O_2_ | 132.0766:0.62:P | 66.93 (16.58) | 9.7 | 100 |
| 15 | **Isoleucine** | C_6_H_13_NO_2_ | 132.1017:0.78:P | 82.93 (22.05) | 9.8 | 100 |
| 16 | **Leucine** | C_6_H_13_NO_2_ | 132.1017:0.79:P | 98.83 (22.90) | 4 | 100 |
| 17 | **Asparagine** | C_4_H_8_N_2_O_3_ | 133.0573:0.84:P | 108.05 (19.19) | 8.3 | 100 |
| 18 | **Ornithine** | C_5_H_12_N_2_O_2_ | 133.0969:0.34:P | 148.88 (46.12) | 14.1 | 100 |
| 19 | **Aspartic acid** | C_4_H_7_NO_4_ | 134.044:0.93:P | 47.99 (11.38) | 8 | 100 |
| 20 | **Hypoxanthine** | C_5_H_4_N_4_O | 137.0459:1.12:P | 48.42 (49.58) | 8.2 | 100 |
| 21 | **Proline betaine** | C_7_H_13_NO_2_ | 144.0988:0.91:P | 14.82 (25.05) | 6.6 | 100 |
| 22 | **Glutamine** | C_5_H_10_N_2_O_3_ | 147.0761:0.87:P | 498.95 (81.99) | 2.6 | 100 |
| 23 | **Lysine** | C_6_H_14_N_2_O_2_ | 147.1128:0.34:P | 146.07 (35.41) | 14.6 | 100 |
| 24 | **Glutamic acid** | C_5_H_9_NO_4_ | 148.0603:0.89:P | 144.44 (41.35) | 4.8 | 100 |
| 25 | **Methionine** | C_5_H_11_NO_2_S | 150.0583:0.85:P | 23.34 (7.06) | 5.8 | 100 |
| 26 | **Histidine** | C_6_H_9_N_3_O_2_ | 156.0766:0.41:P | 81.05 (14.60) | 12.8 | 100 |
| 27 | **Aminooctanoic acid** | C_8_H_17_NO_2_ | 160.1332:0.57:P | 2.57 (0.95) | 8.2 | 100 |
| 28 | **Unknown 2** | C_7_H_16_N_2_O_2_ | 161.1281:0.37:P | 0.15 (0.09)* | 12.3 | 100 |
| 29 | **Carnitine** | C_7_H_15_NO_3_ | 162.1123:0.59:P | 30.66 (7.45) | 8.7 | 100 |
| 30 | **Phenylalanine** | C_9_H_11_NO_2_ | 166.086:0.89:P | 92.81 (14.64) | 3.9 | 100 |
| 31 | **Methylhistidine** | C₇H₁₁N₃O₂ | 170.0922:0.44:P | 22.62 (7.68) | 12.4 | 100 |
| 32 | **Arginine** | C_6_H_14_N_4_O_2_ | 175.1191:0.38:P | 43.35 (11.97) | 9.9 | 100 |
| 33 | **Citrulline** | C_6_H_13_N_3_O_3_ | 176.1025:0.91:P | 29.79 (5.15) | 3.5 | 100 |
| 34 | **Tyrosine** | C_9_H_11_NO_3_ | 182.081:0.94:P | 65.87 (15.54) | 4.7 | 100 |
| 35 | **Asymmetric dimethylarginine** | C_8_H_18_N_4_O_2_ | 203.1499:0.46:P | 1.38 (1.15) | 21.8 | 96 |
| 36 | **Symmetric dimethylarginine** | C_8_H_18_N_4_O_2_ | 203.1499:0.47:P | 1.09 (0.35) | 20 | 96 |
| 37 | **Acetylcarnitine** | C_9_H_17_NO_4_ | 204.1233:0.66:P | 7.16 (2.31) | 7.6 | 100 |
| 38 | **Tryptophan** | C_11_H_12_N_2_O_2_ | 205.0966:0.9:P | 10.55 (3.22) | 9.8 | 100 |
| 39 | **Propionylcarnitine** | C_10_H_19_NO_4_ | 218.1385:0.68:P | 0.95 (0.33) | 19.1 | 97.3 |
| 40 | **Cystine** | C_6_H_12_N_2_O_4_S_2_ | 241.02989:0.9:P | 32.60 (5.29) | 4.1 | 100 |
| 41 | **Unknown 3** | C_10_H_17_N_3_O_6_ | 276.1191:1.14:P | 0.08 (0.03)* | 10 | 100 |
| 42 | **Cysteinylglycine disulfide** | C_8_H_15_N_3_O_5_S_2_ | 298.0526:0.72:P | 0.12 (0.03)* | 4.6 | 100 |
| 43 | **Lactic acid** | C_3_H_6_O_3_ | 89.0244:1.93:N | 5491.94 (1783.43) | 6.9 | 100 |
| 44 | **2-Hydroxyvaleric acid** | C_5_H_10_O_3_ | 117.0557:1.49:N | 0.41 (0.12)* | 13.9 | 100 |
| 45 | **Taurine** | C_2_H_7_NO_3_S | 124.0074:1.02:N | 3.21 (0.77)* | 11.2 | 100 |
| 46 | **Oxoproline** | C_5_H_7_NO_3_ | 128.0353:1.64:N | 25.33 (11.30) | 16.4 | 100 |
| 47 | **3-Methyl-2-oxovaleric acid** | C_6_H_10_O_3_ | 129.0557:1.65:N | 13.99 (5.38) | 15.7 | 100 |
| 48 | **Unknown 4** | - | 130.0871:0.65:N | 3.48 (1.00)* | 6.4 | 100 |
| 49 | **Unknown 5** | - | 145.0614:0.77:N | 5.80 (1.17)* | 9.9 | 100 |
| 50 | **Uric acid** | C_5_H_4_N_4_O_3_ | 167.0211:1.51:N | 370.25 (92.20) | 9.5 | 100 |
| 51 | **Glucose** | C₆H₁₂O₆ | 179.0561:0.58:N | 5270.01 (1144.09) | 2.9 | 100 |
| Metabolites annotated based on their metabolite ID, molecular formula, accurate mass (*m/z*), relative migration time (RMT), ionization mode (P = positive mode, N = negative mode), mean (SD) serum concentration (*mean relative peak area (RPA) in case of unknowns), technical precision of repeated Quality Control samples (QCs) analyzed in each run, and % data completeness. | | | | | | |
