# Supplementary material for: Early sex-dependent differences in metabolic profiles of overweight and adiposity in young children: a cross-sectional analysis
Source: BMC Med. 2023 May 9;21:176. doi: 10.1186/s12916-023-02886-8 (PMC10166631; doi:10.1186/s12916-023-02886-8)
Supplement: Supplementary file 3 — Additional file 3: Table S2. FAMILY baseline characteristics. [file 12916_2023_2886_MOESM3_ESM.docx]

| Table S2: Baseline Characteristics of the FAMILY study population | | | |
| --- | --- | --- | --- |
| Characteristic | **Cases**  **(n=106)** | **Non-Cases**  **(n=350)** | **All Children**  **(n=456)** |
| Age (years) | 5.14 (0.29) | 5.15 (0.28) | 5.15 (0.29) |
| Sex (Female) | 53 (50%) | 177 (51%) | 230 (50.4%) |
| Daily night sleep (hours) | 10.86 (0.55) | 10.93 (0.65) | 10.90 (0.63) |
| Diet quality score | -0.83 (3.45) | -1.13 (3.39) | -1.06 (3.4) |
| Maternal education (years) | 17.44 (3.4) | 17.48 (3.03) | 17.47 (3.11) |
| Social disadvantage index  (Low 0-1)  (Moderate 2-3)  (High 4-5) | 78(76.5%)  19 (18.6%)  5 (4.9%) | 249 (73.7%)  72 (21.3%)  17 (5.0%) | 327 (74.0%)  91 (21.0%)  22 (5.0%) |
| Screen time exposure  Low exposure (<2hours)  High exposure (≥2 hours) | 27 (26%)  78 (74%) | 97 (28%)  248 (72%) | 124 (28.0%)  326 (72.0%) |
| Systolic blood pressure (mm Hg) | 102.85 (7.8) | 98.27 (8.3) | 99.29 (8.40) |
| Diastolic blood pressure (mm Hg) | 61.49 (5.05) | 59.82 (5.62) | 60.21 (5.51) |
| BMI (kg/m^2^) | 18.03 (1.78) | 15.26 (0.86) | 15.88 (1.62) |
| BMI-for-age z-score (WHO) | 1.65 (0.83) | -0.02 (0.62) | 0.35 (0.97) |
| Waist circumference (cm) | 56.37 (4.39) | 50.29 (2.67) | 51.65 (4.03) |
| Waist circumference-to-height | 0.5 (0.04) | 0.46 (0.03) | 0.47 (0.03) |
| Sum of skinfolds (mm) | 24.26 (7.41) | 16.96 (4.15) | 18.59 (5.91) |

Values are presented as mean (SD) or n (%). Diet quality score: sum of daily servings of “healthy” foods less the sum of daily servings of “unhealthy” foods.
