# Supplementary material for: Early sex-dependent differences in metabolic profiles of overweight and adiposity in young children: a cross-sectional analysis
Source: BMC Med. 2023 May 9;21:176. doi: 10.1186/s12916-023-02886-8 (PMC10166631; doi:10.1186/s12916-023-02886-8)
Supplement: Supplementary file 4 — Additional file 4: Table S3. Multiple imputation analysis. [file 12916_2023_2886_MOESM4_ESM.docx]

| Table S3: Serum metabolites associated with child overweight/adiposity at age 5 years from multiple imputed models(n=20) in CHILD* | | | | | |
| --- | --- | --- | --- | --- | --- |
| Metabolite | OR | 95% CI | p- | FDR  d=0.1 | FDR  d=0.05 |
| Threonine | 1.34 | (1.14-1.57) | 0.0004 | 0.009 | 0.005 |
| Leucine | 1.30 | (1.11-1.53) | 0.001 | 0.018 | 0.009 |
| BCAAs | 1.28 | (1.09-1.5) | 0.002 | 0.027 | 0.013 |
| Valine | 1.28 | (1.09-1.49) | 0.002 | 0.036 | 0.018 |
| Glutamic acid | 1.35 | (1.1-1.64) | 0.0028 | 0.045 | 0.023 |
| AAAs | 1.27 | (1.08-1.49) | 0.0034 | 0.054 | 0.027 |
| Phenylalanine | 1.28 | (1.09-1.52) | 0.0032 | 0.063 | 0.032 |
| Glutamine/Glutamic acid | 0.77 | (0.65-0.92) | 0.0049 | 0.072 | 0.036 |
| Isoleucine | 1.22 | (1.04-1.43) | 0.0125 | 0.082 | 0.04 |
| Tyrosine | 1.2 | (1.03-1.4) | 0.020 | 0.09 | 0.045 |
| Oxoproline | 1.2 | (1.02-1.41) | 0.030 | 0.1 | 0.05 |
| *Multivariable logistic regression model adjusting for maternal education, child sleep time, screen time exposure, sex, and age; n=900 following multiple imputations of 20 datasets (n=20). Pooled estimates for OR: odds ratio, 95% CI: confidence intervals, and p-: p-value for statistical significance. FDR: false discovery rate d; when p-value is smaller than this value, association passes multiple hypothesis testing. | | | | | |
