# Supplementary material for: Early sex-dependent differences in metabolic profiles of overweight and adiposity in young children: a cross-sectional analysis
Source: BMC Med. 2023 May 9;21:176. doi: 10.1186/s12916-023-02886-8 (PMC10166631; doi:10.1186/s12916-023-02886-8)
Supplement: Supplementary file 5 — Additional file 5: Table S4. Metabolomics of waist circumference. [file 12916_2023_2886_MOESM5_ESM.docx]

| Table S4. Serum metabolites associated with child waist circumference at age 5 years* | | | | | |
| --- | --- | --- | --- | --- | --- |
| Metabolite | Estimate | Std. Error | z value | p- | 95% CI |
| Alanine | 0.74 | 0.2 | 3.78 | <0.0001 | (0.36-1.12) |
| Proline | 0.71 | 0.19 | 3.77 | <0.0001 | (0.34-1.08) |
| Threonine | 0.74 | 0.19 | 3.88 | <0.0001 | (0.37-1.12) |
| Asparagine | 0.67 | 0.19 | 3.56 | <0.0001 | (0.3-1.04) |
| Glutamic acid | 0.71 | 0.19 | 3.67 | <0.0001 | (0.33-1.09) |
| Phenylalanine | 0.71 | 0.19 | 3.72 | <0.0001 | (0.33-1.08) |
| AAAs | 0.73 | 0.19 | 3.89 | <0.0001 | (0.36-1.1) |
| Valine | 0.64 | 0.19 | 3.41 | 0.001 | (0.27-1) |
| Leucine | 0.61 | 0.19 | 3.21 | 0.001 | (0.24-0.98) |
| Tyrosine | 0.6 | 0.19 | 3.21 | 0.001 | (0.23-0.97) |
| Glutamine/Glutamic acid | -0.64 | 0.19 | -3.32 | 0.001 | (-1.02--0.26) |
| Oxoproline | 0.61 | 0.19 | 3.13 | 0.002 | (0.23-0.99) |
| BCAAs | 0.58 | 0.19 | 3.08 | 0.002 | (0.21-0.95) |
| Lactic acid | 0.55 | 0.2 | 2.81 | 0.005 | (0.17-0.93) |
| Choline | 0.53 | 0.2 | 2.69 | 0.007 | (0.14-0.91) |
| Serine | 0.52 | 0.19 | 2.72 | 0.007 | (0.15-0.9) |
| Isoleucine | 0.47 | 0.19 | 2.46 | 0.014 | (0.09-0.84) |
| Aspartic acid | 0.49 | 0.2 | 2.47 | 0.014 | (0.1-0.87) |
| Unknown 276.1191 | -0.48 | 0.2 | -2.46 | 0.014 | (-0.86--0.1) |
| Ornithine | 0.49 | 0.2 | 2.45 | 0.015 | (0.1-0.88) |
| Glucose | -0.48 | 0.2 | -2.43 | 0.015 | (-0.87--0.09) |
| Hypoxanthine | 0.45 | 0.2 | 2.27 | 0.023 | (0.06-0.84) |
| Unknown 161.1281 | 0.44 | 0.2 | 2.26 | 0.024 | (0.06-0.83) |
| 2-Hydroxyvaleric acid | 0.39 | 0.19 | 2.07 | 0.038 | (0.02-0.76) |
| Arginine | -0.4 | 0.2 | -2.01 | 0.045 | (-0.78--0.01) |
| *Multivariable linear regression model adjusting for maternal education, child sleep time, breastfeeding status at 1 year, sex, and age [204 (23%) had missing values on at least one covariate; complete cases analysis n=696: 173 cases and 523 controls]. | | | | | |
