# Supplementary material for: Early sex-dependent differences in metabolic profiles of overweight and adiposity in young children: a cross-sectional analysis
Source: BMC Med. 2023 May 9;21:176. doi: 10.1186/s12916-023-02886-8 (PMC10166631; doi:10.1186/s12916-023-02886-8)
Supplement: Supplementary file 6 — Additional file 6: Table S5. Metabolomics of body mass index. [file 12916_2023_2886_MOESM6_ESM.docx]

| Table S5. Serum metabolites associated with child z-BMI at age 5 years* | | | | | |
| --- | --- | --- | --- | --- | --- |
| Metabolite | Estimate | Std. Error | z value | p- | 95% CI |
| Alanine | 0.114 | 0.036 | 3.139 | 0.002 | (0.043-0.185) |
| 2-Hydroxyvaleric acid | 0.101 | 0.035 | 2.925 | 0.004 | (0.033-0.169) |
| Proline | 0.093 | 0.035 | 2.651 | 0.008 | (0.024-0.162) |
| Aminooctanoic acid | 0.085 | 0.035 | 2.433 | 0.015 | (0.016-0.153) |
| Carnitine | 0.082 | 0.036 | 2.261 | 0.024 | (0.011-0.154) |
| Creatinine | 0.076 | 0.036 | 2.131 | 0.033 | (0.006-0.145) |
| Threonine | 0.071 | 0.036 | 1.982 | 0.048 | (0.001-0.141) |
| *Multivariable linear regression model adjusting for maternal education, child sleep time, breastfeeding status at 1 year, sex, and age [204 (23%) had missing values on at least one covariate; complete cases analysis n=696: 173 cases and 523 controls]. | | | | | |
