# Supplementary material for: Early sex-dependent differences in metabolic profiles of overweight and adiposity in young children: a cross-sectional analysis
Source: BMC Med. 2023 May 9;21:176. doi: 10.1186/s12916-023-02886-8 (PMC10166631; doi:10.1186/s12916-023-02886-8)
Supplement: Supplementary file 7 — Additional file 7: Table S6. Metabolomics of sum of skinfolds. [file 12916_2023_2886_MOESM7_ESM.docx]

| Table S6. Serum metabolites associated with child sum of skinfolds at age 5 years* | | | | | |
| --- | --- | --- | --- | --- | --- |
| Metabolite | Estimate | Std. Error | z value | p- | 95% CI |
| Valine | 0.536 | 0.174 | 3.088 | 0.002 | (0.195-0.877) |
| AAAs | 0.479 | 0.176 | 2.72 | 0.007 | (0.133-0.826) |
| Propionylcarnitine | 0.511 | 0.191 | 2.674 | 0.008 | (0.136-0.887) |
| BCAAs | 0.468 | 0.177 | 2.649 | 0.008 | (0.121-0.815) |
| Tyrosine | 0.457 | 0.174 | 2.626 | 0.009 | (0.115-0.798) |
| Leucine | 0.444 | 0.177 | 2.515 | 0.012 | (0.097-0.791) |
| Isoleucine | 0.443 | 0.178 | 2.486 | 0.013 | (0.093-0.792) |
| Phenylalanine | 0.411 | 0.179 | 2.298 | 0.022 | (0.06-0.762) |
| Dimethylglycine | 0.371 | 0.18 | 2.06 | 0.04 | (0.017-0.724) |
| Glutamic acid | 0.369 | 0.182 | 2.029 | 0.043 | (0.012-0.726) |
| Cystine | 0.364 | 0.179 | 2.029 | 0.043 | (0.012-0.716) |
| Serine | 0.356 | 0.181 | 1.964 | 0.05 | (0-0.712) |
| *Multivariable linear regression model adjusting for maternal education, child sleep time, breastfeeding status at 1 year, sex, and age [204 (23%) had missing values on at least one covariate; complete cases analysis n=696: 173 cases and 523 controls]. | | | | | |
