# Supplementary material for: Early sex-dependent differences in metabolic profiles of overweight and adiposity in young children: a cross-sectional analysis
Source: BMC Med. 2023 May 9;21:176. doi: 10.1186/s12916-023-02886-8 (PMC10166631; doi:10.1186/s12916-023-02886-8)
Supplement: Supplementary file 8 — Additional file 8: Table S7. Results of the FAMILY validation cohort. [file 12916_2023_2886_MOESM8_ESM.docx]

| Table S7: Serum metabolites associated with child overweight/adiposity at age 5 years* in FAMILY | | | | |
| --- | --- | --- | --- | --- |
| Metabolite | OR | 95% CI | p- | FDR |
| Tyrosine | 1.44 | (1.14-1.82) | 0.002 | 0.017 |
| AAAs | 1.43 | (1.13-1.81) | 0.003 | 0.033 |
| Glutamine/Glutamic acid | 0.71 | (0.56-0.89) | 0.003 | 0.05 |
| Leucine | 1.35 | (1.07-1.71) | 0.011 | 0.067 |
| Glutamic acid | 1.33 | (1.05-1.68) | 0.016 | 0.083 |
| Phenylalanine | 1.27 | (1.01-1.6) | 0.04 | 0.1 |
| Isoleucine | 1.25 | (1-1.57) | 0.05 |  |
| BCAAs | 1.23 | (0.97-1.56) | 0.08 |  |
| Threonine | 1.13 | (0.9-1.43) | 0.30 |  |
| Oxoproline | 0.93 | (0.73-1.17) | 0.52 |  |
| Valine | 1.04 | (0.82-1.34) | 0.72 |  |
| OR:odds ratio, 95% CI: confidence intervals, p-: p-value for statistical significance, FDR: false discovery rate d=0.1; when p- is smaller than this value, association passes multiple hypothesis testing.  *Multivariable logistic regression model adjusting for maternal education, child sleep time, breastfeeding status at 1 year, sex, and age [36 (8%) had missing values on at least one covariate; complete cases analysis n=420: 96 cases and 324 controls]. | | | | |
